# Network Analysis Identifies Microsomal Glutathione S‐Transferase as a Potential Regulator of Oxidative Stress and Proteasome Dysfunction in Human Osteoarthritic Menisci

**DOI:** 10.1096/fba.2025-00302

**Published:** 2026-04-27

**Authors:** Xinjie Wu, Amina Hamzatova, Cecilia Aulin, Wei Sun, Andre Struglics, David A. Hart, Paul W. Ackermann, Aisha S. Ahmed

**Affiliations:** ^1^ Department of Orthopaedic Surgery, Nanjing Drum Tower Hospital The Affiliated Hospital of Nanjing University Medical School Nanjing Jiangsu People's Republic of China; ^2^ Department of Molecular Medicine and Surgery Center for Molecular Medicine Stockholm Sweden; ^3^ Department of Medicine Center for Molecular Medicine Stockholm Sweden; ^4^ Department of Orthopaedic Surgery of the Perelman School of Medicine University of Pennsylvania Philadelphia Pennsylvania USA; ^5^ Orthopaedics, Department of Clinical Sciences Lund, Faculty of Medicine Lund University Lund Sweden; ^6^ Department of Surgery, Faculty of Kinesiology and the McCaig Institute for Bone & Joint Health University of Calgary Calgary Canada

**Keywords:** meniscus, microsomal glutathione S‐transferase, osteoarthritis, ubiquitin‐proteasome system, weighted gene co‐expression network analysis

## Abstract

Osteoarthritis (OA)‐related meniscal degeneration involves complex interactions between oxidative stress and proteasomal dysfunction. However, the molecular drivers of regional meniscal vulnerability remain poorly defined. This study integrated multiple transcriptomic datasets from OA and control menisci to identify functional networks and hub genes by using weighted gene co‐expression network analysis. Human meniscal tissues from medial and lateral compartments were harvested during total knee arthroplasty and subjected to western blot analysis. In vitro assays on the basis of human chondrocytes were exposed to lipopolysaccharide or the proteasome inhibitor MG132 (carbobenzoxy‐l‐leucyl‐l‐leucyl‐l‐leucinal) to evaluate the stimulus‐specific regulation of identified network and hub genes. Weighted gene co‐expression network analysis revealed microsomal glutathione S‐transferase (MGST1) as the hub gene within a module enriched for ubiquitination and proteasome activity. Experimental validation in human meniscal tissues demonstrated pronounced upregulation of MGST1, ubiquitin‐conjugating enzyme E2 N (UBE2N), and proteasome activator complex subunit alpha (PSMA) in mechanically overloaded medial compartments compared to lateral regions. In vitro studies demonstrated stimulus‐specific modulation: lipopolysaccharide‐induced inflammatory stress upregulated MGST1, whereas proteasome inhibition via MG132 led to its downregulation. These findings highlight a dynamic interplay between redox adaptation and proteostasis, where chronic mechanical stress drives MGST1‐mediated antioxidant responses and compensatory ubiquitination. Together, these results suggest that joint tissues dynamically adapt to mechanical and inflammatory challenges by modulating oxidative stress defenses and protein quality control mechanisms, processes central to OA pathophysiology.

## Graphical abstract

Schematic diagram of the study design. OA = osteoarthritis, GSE = Genomic Spatial Event database, DEG = Differentially expressed genes, WGCNA = Weighted gene co‐expression network analysis, MCC = Maximal clique centrality.

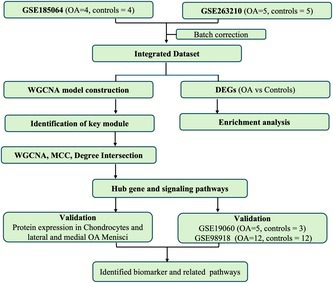

## 1 Introduction

Osteoarthritis (OA), a leading cause of disability worldwide, is characterized by progressive degradation of articular cartilage and associated joint tissues, including the meniscus.

Although chronic mechanical stress and oxidative damage are recognized drivers of OA pathogenesis, the molecular mechanisms underlying regional meniscal vulnerability remain poorly understood [1]. The meniscus is essential for load distribution and stability of the knee joint. OA leads to extracellular matrix disruption and cellular senescence, resulting in reduced cartilage thickness and meniscal stiffness. These changes can cause meniscal displacement and narrowing, which increase abnormal mechanical stress and accelerate cartilage degradation, particularly in the medial compartment [2]. Recent studies indicate that oxidative stress‐related pathways, including ferroptosis and inflammatory signaling, exacerbate chondrocyte injury, whereas modulation of mechanobiological processes and redox homeostasis can mitigate OA progression by enhancing cartilage regeneration and osteogenic differentiation [3, 4]. Moreover, emerging evidence highlights the interplay between ubiquitin‐proteasome system dysregulation and redox imbalance in OA progression [5, 6].

The ubiquitin‐proteasome system, responsible for targeted protein degradation, is tightly regulated under physiological conditions but becomes dysregulated under oxidative stress, leading to the accumulation of damaged proteins and cellular dysfunction. Key components such as ubiquitin‐conjugating enzymes and proteasome subunits have been implicated in OA cartilage degradation [7]. However, their involvement in meniscal degeneration is underexplored. Concurrently, oxidative stress markers, including lipid peroxidation products, are elevated in OA joints [8, 9], prompting compensatory upregulation of antioxidant enzymes. However, whether these pathways interact spatially or functionally in the meniscus remains to be characterized.

The aim of this study was to explore molecular mechanisms underlying OA by applying weighted gene co‐expression network analysis (WGCNA) to publicly available transcriptomic data from meniscal tissue, with the goal of identifying co‐expressed gene modules and hub genes linked to oxidative stress and proteasomal regulation. The WGCNA identified microsomal glutathione S‐transferase (MGST1) as a hub gene within the module enriched for ubiquitination and proteasome activity in this study. Experimental validation in human meniscal tissues and LPS‐activated chondrocytes revealed regional and stress‐specific dysregulation of MGST1, ubiquitin‐conjugating enzyme E2 N (UBE2N), and proteasome activator complex subunit alpha (PSMA), highlighting their roles in mitigating oxidative damage and maintaining proteostasis. These findings advance our understanding of molecular mechanisms in OA menisci and provide novel insights into therapeutic strategies.

## 2 Materials and Methods

### 2.1 Data Acquisition and Preprocessing

Gene expression profiles for meniscal OA and control samples were obtained from the Gene Expression Omnibus (GEO) database, specifically datasets GSE185064 [10] and GSE263210 [11]. Two additional publicly available datasets, GSE19060 and GSE98918, were downloaded from the GEO database for validation. The study design is shown in the Graphical Abstract Image and details of datasets reported in Table S1. Batch effects between the datasets were adjusted using the ComBat function from the sva package [12]. The correction was validated through boxplots, density plots, and Principal Component Analysis (PCA), ensuring sample distribution was normalized and batch‐associated variance was minimized.

### 2.2 Differential Expression and Functional Enrichment Analysis

Differentially expressed genes (DEGs) between OA and control samples from menisci were identified using the limma package. A threshold of adjusted *p* < 0.05 and |log_2_FC| > 1.2 was applied to define significant DEGs. Visualization of DEGs was performed through volcano plots and heatmaps. To explore the biological significance of DEGs, Gene Ontology (GO) and Reactome pathway analyses were conducted using the clusterProfiler packages [13].

### 2.3 Co‐Expression Network Construction and Hub Gene Identification

Weighted Gene Co‐expression Network Analysis (WGCNA) was applied to the combined expression matrix to identify gene modules associated with OA [14]. A soft‐thresholding power of 9 was chosen to ensure scale‐free topology. Module‐trait relationships were then assessed by correlating module eigengenes with clinical and sample traits. Significant modules were identified on the basis of *P* value < 0.05. To avoid false positives, hub genes are defined as the top 10% genes with the highest degree that have module membership (MM) > 0.8. Furthermore, hub genes were then identified through an integrative approach combining the Maximal clique centrality (MCC) and Degree algorithms (via Cytoscape) with hub genes from WGCNA.

### 2.4 Validation in Independent Datasets

To validate the expression pattern of MGST1, one of the hub genes identified in the integrative analysis, two publicly available datasets, GSE19060 and GSE98918, were downloaded from the GEO database. Both datasets include transcriptomic profiles of human meniscus tissues from OA patients and controls, comprising 17 OA and 15 control samples in total (GSE19060: OA = 5, Control = 3 [15]; GSE98918: OA = 12, Control = 12 [16], Table S1). Gene expression matrices were normalized as per platform‐specific guidelines. The hub gene expression levels were extracted and statistically compared between OA and control groups. A *p*‐value < 0.05 was considered statistically significant.

### 2.5 Subjects and Sampling for Protein Verification

A total of seven patients (Median age 66 years (56–79 years), 5 males, 2 females) with OA undergoing total knee arthroplasty were included in the study (Table S2). All patients were diagnosed according to the American College of Rheumatology criteria for OA [17]. All participants were informed about the study procedure and written consent was obtained. Knee joint meniscus tissues from the medial and lateral sides were collected during surgery and immediately frozen at −80°C for future analysis. Patient characteristics are presented in Table S1. The study was conducted after approval from the Regional Ethical Review Committee in Sweden (Dnr 2013–598) and followed all guidelines according to the Declaration of Helsinki.

### 2.6 In Vitro Chondrocyte Stimulation and Expression Analysis

Human chondrocyte cell line C28/I2 was obtained from the American Type Culture Collection (ATCC) and cultured in Dulbecco's Modified Eagle Medium (DMEM) supplemented with 10% fetal bovine serum (FBS, Gibco) and 1% penicillin–streptomycin (PEST, Gibco) at 37°C in an incubator with a humidified atmosphere containing 5% CO2. Chondrocytes were used in the experiments because of the unavailability of primary meniscal cells and the fact that the inner meniscal region possesses a type II collagen–rich extracellular matrix, a cellular phenotype resembling that of articular cartilage [18]. Cells were dissociated with 0.25% trypsin‐ethylenediaminetetraacetic acid (Trypsin–EDTA) (ThermoFisher Scientific) and seeded in 12‐well plates with 2.5 × 10^5^ cells/well. Cells were treated with lipopolyscharide (LPS, 1 μg/mL) to mimic inflammatory stimulation and carbobenzoxy‐l‐leucyl‐l‐leucyl‐l‐leucinal MG132 (10 μM) to induce proteasome inhibition for 24 h. Post‐treatment, cells were harvested and subjected to Western blot analysis.

### 2.7 Protein Extraction and Western Blot Analysis

Frozen medial and lateral menisci tissues collected from seven OA patients were powdered using a Mikro‐dismembrator (B. Braun Biotech International, Germany) on dry ice and mixed with protein extraction buffer (Thermo Scientific, Cat no. 8990) containing 10% phosphatase (Sigma, cat no. P2850) and 10% protease inhibitor cocktail (Thermo Scientific, Cat no. P8340). Samples were sonicated at 35% effect while keeping on ice and with short bursts until completely homogenized before being centrifuged at 3000 *g* for 15 min at 4°C. Supernatants corresponding to the protein extracts were collected, aliquoted, and stored at −80°C. Protein content was measured using the BCA Assay Kit (Thermo Scientific, Cat no. 23225).

For Western blot, 10 μg of protein per well was loaded, separated by gel electrophoresis (4%–12% Bis‐Tris, Invitrogen, Cat no. 4561033), and transferred to nitrocellulose membranes as described previously [19]. The membranes were incubated with 10% bovine serum albumin in 1× tris‐buffered saline with 0.1% tween (TBST) to block unspecific binding sites. The membranes were subsequently covered with primary antibodies against MGST1 (0.1 μg/ml), PSMA (0.1 μg/ml), UBE2N, and β‐actin (0.02 μg/ml) (Cell Signaling, Boston, USA) overnight at 4°C. After washing, the membranes were incubated with secondary antibodies anti‐rabbit IgG and/or anti‐mouse IgG conjugated to HRP (Cell Signaling, Boston, USA). The chemiluminescence signals were initiated by using SuperSignal West Femto Maximum Sensitivity Substrate (Thermo Fisher Scientific). Chemiluminescence was identified and quantified by using Biorad ChemiDoc MP Imaging, and analysis was performed in Image Lab. Immunopositive bands were normalized against the bands of β‐actin, and results were presented as fold change from control values.

### 2.8 Antibodies and Reagents

The antibodies used in the present study were as follows: anti‐MGST1 (3,116,640; rabbit mAb, 1:1000), anti‐UBE2N (D2A1; #6999S; rabbit mAb, 1:1000), anti‐β‐actin (13E5; rabbit mAb, 1:1000), and anti‐PSMA (#2455S; rabbit mAb, 1:1000) (Cell Signaling, Boston, USA). All secondary antibodies were purchased from ThermoFisher (Oxford, UK). Proteasome inhibitor MG‐132 (MG132, #2194) was purchased from Cell Signaling (BioNordika, Stockholm, Sweden), and bacterial LPS (L2630) was purchased from Sigma (Steinheim, Germany).

### 2.9 Statistical Analysis

All statistical analyses were performed using R software (version 4.0.4; R Core Team 2020, Vienna, Austria) or IBM SPSS (version 29.0.2.0 (20)). Continuous variables were expressed as the mean ± SD values and compared with the Student's t–test or Mann–Whitney U test. Categorical variables were expressed as frequencies and proportions and compared with the chi‐square and Fisher's exact tests or Kruskal–Wallis test. Correlation coefficients and *p*‐values were computed using the cor.test function or by Spearman's rank correlation coefficient. Data with a *P* value < 0.05 was considered significant.

## 3 Results

### 3.1 Integration of Transcriptomic Datasets Reveals Robust Batch Correction

**FIGURE 1:**
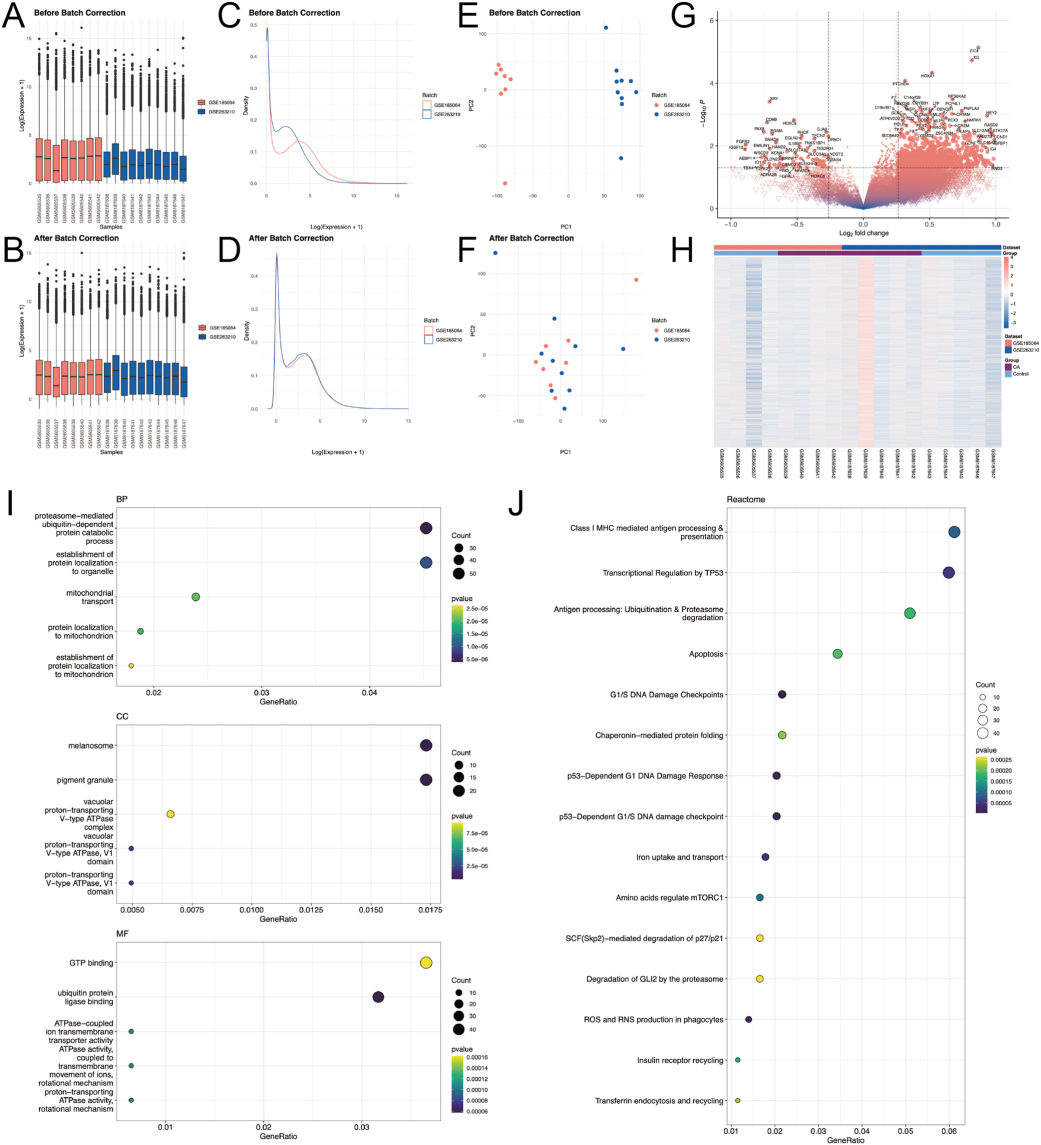
Integration and batch correction of GSE185064 and GSE263210 datasets. (A, B) Boxplots showing gene expression distributions before and after batch effect correction. (C, D) Density plots of log‐transformed expression values before and after batch correction. (E, F) PCA plots illustrating the clustering of samples before and after batch effect removal. (G) Volcano plot of DEGs between OA and control samples. (H) Heatmap of DEGs between OA and control samples. (I) GO enrichment analysis of DEGs, depicting significant biological processes (BP), cellular components (CC), and molecular functions (MF). (J) Reactome pathway analysis of DEGs, showing key enriched pathways related to OA pathophysiology. DEG = Differentially Expressed Genes.

Integrated datasets of GSE185064 and GSE263210 included totally 18 samples, comprising 9 OA and 9 control meniscus tissue samples, with 17,977 shared genes. ComBat‐based normalization effectively minimized batch effects between two datasets, as evidenced by harmonized gene expression distributions (Figure 1A,B) and log‐transformed density profiles (Figure 1C,D). PCA demonstrated reduced inter‐batch variability, with batch‐associated variance decrease (Figure 1E,F). Differential expression analysis identified 893 DEGs. (Figure 1G,H). The GO analysis identified proteasome‐mediated ubiquitin dependent protein processing as the most important biological process (Figure 1I) in addition to protein localization to organelle and in mitochondria. Functional enrichment highlighted MHC mediated antigen presentation, transcriptome regulation by TP53, ubiquitin‐mediated proteolysis, proteasome degradation, and apoptosis as significant pathways (Figure 1J).

### 3.2 WGCNA Identifies OA‐Associated Modules and MGST1 as a Hub Gene

**FIGURE 2:**
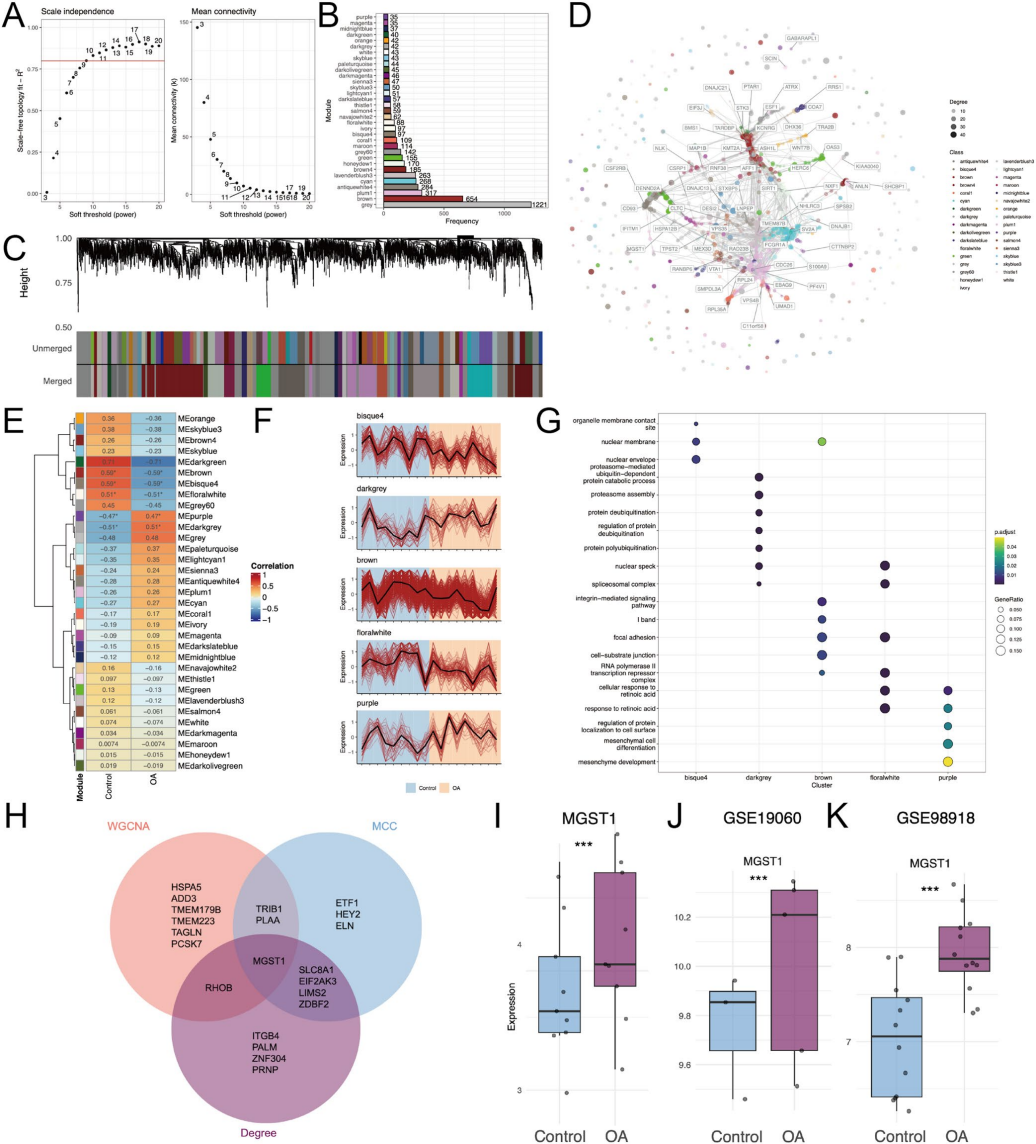
Weighted gene co‐expression network analysis and identification of hub genes. (A) Scale‐free topology analysis for soft‐threshold power selection in WGCNA. (B) Bar plot of module membership, showing the number of genes in each module. (C) Dendrogram displaying hierarchical clustering of genes into co‐expression modules. (D) Gene co‐expression network constructed by WGCNA, highlighting key module associations. (E) Correlation heatmap of gene modules with OA phenotypes, identifying significantly related modules. (F) Module eigengene expression profiles, showing distinct clustering patterns. (G) GO enrichment analysis of the bisque4, dark grey, brown, floralwhite, and purple modules. (H) Venn diagram illustrating the intersection of hub genes identified by WGCNA, MCC, and Degree methods, with MGST1 as the common hub gene. (I) Boxplot showing MGST1 expression levels between control and OA groups. (J, K) Validation of MGST1 expression in independent datasets (GSE19060 and GSE98918), demonstrating consistent upregulation in OA. Significant differences indicated as ***p < 0.001. GO = Gene ontology.

Using the top 5000 most variable genes derived from the integrated dataset, a soft threshold power of β = 9 was selected via scale‐free topology analysis (Figure 2A). Weighted correlation network analysis (WGCNA) delineated 33 co‐expression modules (Figure 2B–D). Among these, five modules, bisque4, dark grey, brown, floralwhite, and purple, correlated strongly with OA (Figure 2E,F). Interestingly, further analysis of these five modules highlighted that the dark grey module enriched proteasome activity and protein deubiquitination (Figure 2G). Intersection of WGCNA hub genes with MCC/Degree rankings pinpointed MGST1, an oxidative stress marker, as the consensus hub gene (Figure 2H) with significantly elevated levels in OA compared to controls (Figure 2I). Cross‐dataset validation further confirmed MGST1 upregulation in OA menisci (Figure 2J,K).

### 3.3 Validation in Meniscal Tissues and Chondrocytes

**FIGURE 3:**
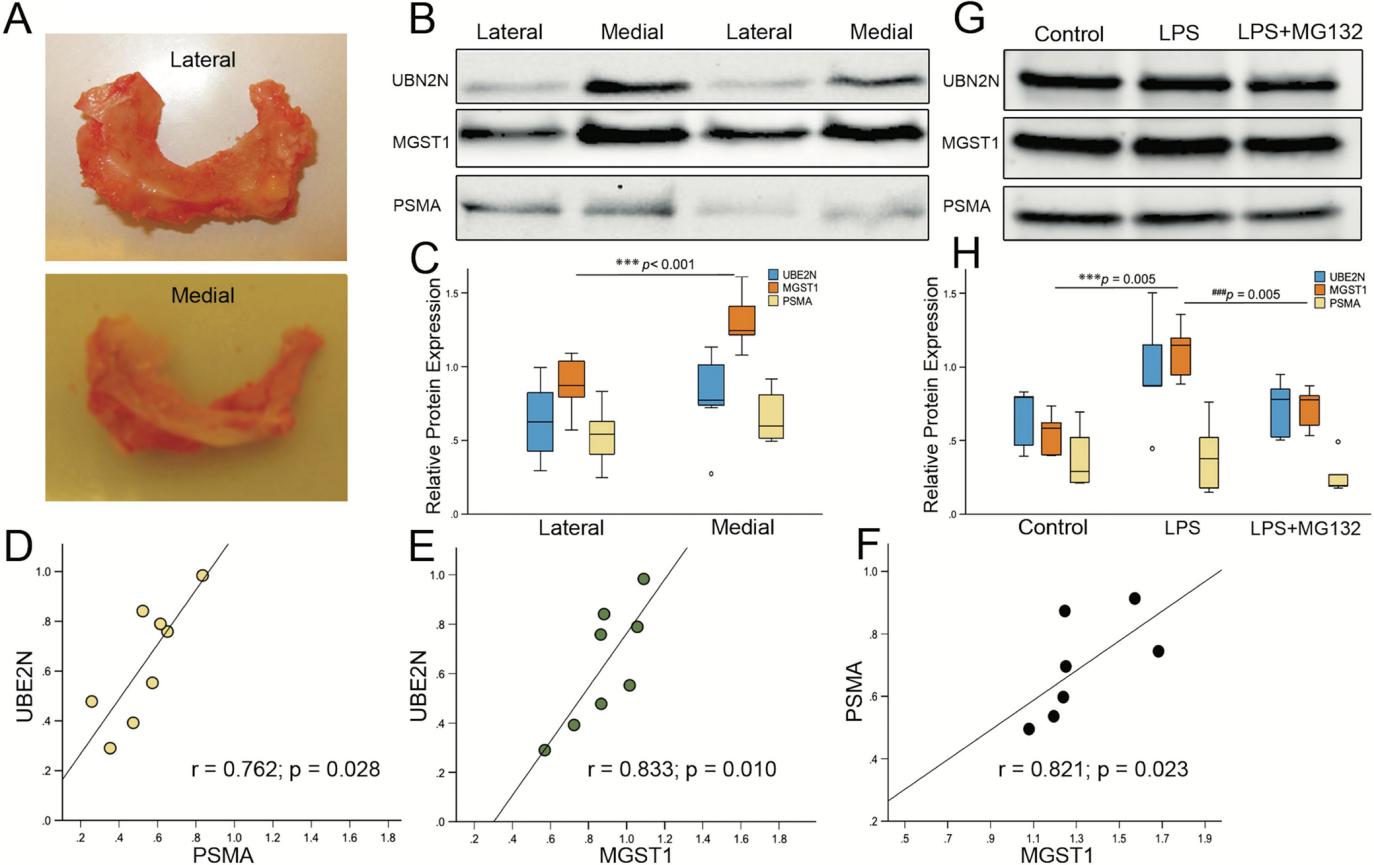
Validation of roles of MGST1 and proteasome systems in meniscus tissues and chondrocytes. Schematic representation of (A) lateral and medial menisci samples collected from knee OA patients. Representative images of western blot analysis (B) and quantitative analysis (C) of UBE2N, MGST1, and PSMA expression in lateral (*n* = 8) and medial (*n* = 7) menisci samples. Results are expressed as a box plot with interquartile range. ***p ≤ 0.01 compared to lateral compartment (Mann–Whitney U test). Association among (D) UBE2N and PSMA, (E) MGST1 and UBE2N in the lateral (*n* = 8; Spearman correlation coefficient), and (F) MGST1 and PSMA in medial menisci (*n* = 7; Spearman correlation coefficient). Western blot analysis (G) and quantitative analysis (H) of UBE2N, MGST1, and PSMA expression in control, lipopolysaccharide (LPS), and LPS + MG132‐stimulated chondrocytes (*n* = 5 technical replicates). Results are expressed as a box plot with interquartile range. ***p ≤ 0.01 compared to control, and ^###^ *p* ≤ 0.01 compared to LPS stimulated (Kruskal–Wallis test).

To dissect the spatial and functional coordination of oxidative stress (MGST1), ubiquitination (UBE2N), and proteolysis (PSMA), the MGST1, ubiquitin‐conjugating enzyme (UBE2N), and the proteasome subunit alpha (PSMA) were concurrently analyzed in OA menisci (Figure 3A,B) and LPS‐stimulated chondrocytes. The medial compartments in OA meniscal tissues exhibited pronounced overexpression of all three genes, with MGST1 significantly elevated compared to lateral regions (Figure 3C). UBE2N and PSMA also trended higher medially, though without statistical significance (Figure 3C). In the lateral menisci, positive associations were observed between UBE2N vs. PSMA and vs. MGST1 (Figure 3D,E), whereas in medial menisci among PSMA and MGST1 (Figure 3F) reinforcing their relationships. To test whether inflammatory or proteotoxic stress directly regulates MGST1, chondrocytes were stimulated with LPS (mimicking inflammation) and exposed to MG132 (proteasome inhibition). LPS markedly increased MGST1 protein expression indicating acute inflammation boosts antioxidant defense while maintaining the ubiquitination and proteasomal activity for protein turnover (Figure 3G,H). MG132 treatment, mimicking proteotoxic stress, led to suppressed LPS‐stimulated MGST1 levels, indicating that when the proteasome is pharmacologically stalled, cells compensate by reducing redox buffering without affecting ubiquitination or core proteasome subunits.

## 4 Discussion

This study integrated multiple datasets and experimental validation to investigate the molecular mechanisms underlying OA‐associated meniscal degeneration. The findings highlight the critical role of the glutathione transferase MGST1 in mediating oxidative stress responses and proteasomal regulation, with spatial and stress‐dependent expression patterns that align with OA degenerative changes. The coordinated dysregulation of MGST1, UBE2N, and PSMA underscores the interplay between redox homeostasis and protein degradation in OA pathophysiology.

The pronounced upregulation of MGST1 in mechanically stressed medial menisci of OA patients suggests its role as a compensatory antioxidant mediator. Mechanical overload in the knee joint generates reactive oxygen species (ROS), which can contribute to extracellular matrix degradation and cellular senescence [20, 21]. MGST1, known to detoxify lipid peroxidation products [22], may protect meniscal fibrochondrocytes from oxidative damage, particularly in high‐load regions. This regional specificity aligns with studies showing spatial heterogeneity in redox enzyme expression within OA‐affected joints [23]. The data reveal and characterize a previously underexplored stress‐response axis comprising UBE2N, MGST1, and PSMA in OA.

These results provide evidence for a tightly regulated interplay between ubiquitination, antioxidant defense, and proteasomal degradation, three central processes that together contribute to cellular homeostasis under inflammatory and proteotoxic stress. UBE2N facilitates K63‐linked ubiquitination, a process critical for NF‐κB activation and inflammatory signaling, whereas MGST1 simultaneously augments glutathione‐mediated detoxification to counteract oxidative by‐products of inflammation [24, 25]. PSMA upregulation indicates an enhanced proteasomal activity able to clear ubiquitinated substrates [26, 27]. Although each component may independently confer short‐term protection, their **sustained co‐activation** may perpetuate a detrimental feedback loop by maintaining inflammatory signaling with heightened proteasomal activity contributing to progressive tissue degeneration as observed in medial menisci. Interestingly, the elevated MGST1 as observed in this study is in line with a recent observation that identified MGST1 as a clinical biomarker for knee OA, demonstrating that its elevated expression in the infrapatellar fat pad correlates with both the severity of pre‐operative clinical symptoms and the patient's response to treatment [28].

The observation of markedly upregulated MGST1 and a strong association with PSMA in the medial compartment likely reflects a coordinated cellular response at the potentially overloaded compartment to resolve inflammation by reducing oxidative burden and clearing damaged proteins. Interestingly, the upregulation of MGST1 in OA menisci was also confirmed in two independent RNA‐seq datasets of meniscal tissue. These consistent findings reinforce our assumption that oxidative stress processes are subject to mechanical regulation, a mechanism that may play a critical role in the pathogenesis of OA by linking altered joint mechanics to redox imbalance, inflammation, and tissue degeneration. Consistent with this assumption are the comparative results of the present in vitro experiments that revealed unique regulatory patterns under acute inflammatory (LPS) versus chronic proteotoxic (MG132) stress. That is, exposure to LPS led to an upregulated MGST1 as well as UBE2N protein expression while maintaining the PSMA, suggesting that acute inflammation accelerates ubiquitination and antioxidant defenses. This synergy suggests that inflammation triggers a dual activation of redox and proteolytic pathways, which attempt to protect chondrocytes from damage. Such coordination may be particularly relevant in early OA, where episodic inflammation initiates tissue remodeling and pain sensitization; however, these findings implicate a sustained activity even in advanced stages of OA, potentially contributing to tissue adaptation or degeneration. This dual activation potentially contributes to exacerbated cellular dysfunction and tissue degeneration, aligning with features of late‐stage OA.

The in vitro experiments also revealed distinct regulatory patterns under acute inflammatory (LPS) versus chronic proteotoxic (MG132) stress. LPS stimulation led to a marked upregulation of MGST1, indicative of an amplified redox defense. In contrast, treatment with MG132, a proteasome inhibitor stimulating chronic proteotoxic stress, resulted in a significant reduction in MGST1. These findings highlight a tightly coordinated balance between oxidative stress responses and proteasome system activity, essential for cellular homeostasis in OA cartilage. The observed downregulation suggests that proteasome inhibition disrupts compensatory stress response pathways, possibly because of feedback suppression or cellular exhaustion. MG132 treatment may induce the accumulation of misfolded proteins and ubiquitinated substrates, which can overwhelm the cellular machinery and lead to transcriptional reprogramming or silencing of protective genes. These findings underscore the context‐dependent nature of stress‐response genes and highlight the importance of temporal and dose considerations when targeting the proteasome for therapeutic purposes in OA and related pathologies. For in vitro studies, the C28/I2 human chondrocyte cell line was used. Although C28/I2 cells provide a practical and well‐characterized model, they do not fully represent the biomechanical environment, heterogeneity, or transcriptional profile of meniscus fibrochondrocytes. These limitations should be considered when interpreting the translational relevance of the findings, particularly with respect to meniscus‐specific biology.

In line with the bioinformatic analysis, in vivo and in vitro analyses demonstrated that MGST1‐mediated oxidative stress defense was altered under acute inflammatory and proteotoxic stress conditions. Notably, UBE2N and PSMA showed more modest, statistically non‐significant changes; however, the consistent regulation of UBE2N across models points to its potentially important modulatory role. UBE2N is increasingly recognized for its involvement in non‐degradative ubiquitination events that regulate inflammatory signaling and stress responses [29, 30]. This potential role as a signaling hub suggests that even subtle fluctuations in UBE2N expression could have broad implications for cellular adaptation to stress. These findings also align with accumulating evidence that ubiquitination is not only essential for proteasomal degradation but also orchestrates proteotoxic responses through its crosstalk with autophagy and redox regulation [31]. Together, these proteolytic pathways could orchestrate the selective degradation and recycling of intracellular proteins, processes that are essential for maintaining proteostasis, particularly in the setting of sustained mechanical and inflammatory stress.

From a therapeutic perspective, this UBE2N‐MGST1‐PSMA axis presents multiple opportunities for intervention. Selective and reversible proteasome inhibitors may be repurposed at lower doses to fine‐tune proteolytic activity in OA without triggering adverse effects. Similarly, pharmacological modulation of MGST1 or its upstream regulators may mitigate oxidative damage in chronic OA. Notably, the stress‐specific modulation of UBE2N and PSMA suggests that interventions should be tailored to disease stage for optimal effectiveness.

The present study has several limitations. Because of a relatively small number of studies with menisci tissues and a small sample size, only 4 studies were included in the analysis; however, future studies with larger sample size cohorts should be performed to confirm the role of specific hub proteins. Although the in vitro studies supported the bioinformatic findings, more advanced in vivo experiments on the basis of animal models, genetically modified primary cells, or cells cultured under hypoxic conditions mimicking the cartilage and menisci microenvironment should be used to assess the potential role of MGST1 and proteasome on menisci degeneration leading to OA progression. In addition, several signaling pathways are involved in OA disease initiation and/or progression; although the proteasome pathway was selected for further investigation because of its high enrichment score. Nevertheless, other pathways likely should also be further investigated for their contributions, an investigation that may also provide new insights into OA pathology.

In conclusion, the data presented reveal that potential mechanical overload at the medial compartment in OA can drive regional overexpression of MGST1 to counteract oxidative damage, while simultaneously modulating ubiquitination and proteasomal activity to manage protein homeostasis. These findings advance our understanding of OA heterogeneity and identify MGST1 as a potential therapeutic target for preserving meniscal integrity.

## Author Contributions

X. W designed the study layout, bioinformatic and statistical data analysis, interpretation of data, and manuscript writing and reviewing. A. H performed functional experiments and participated in data collection and interpretation. A. S and C. A for providing the patients’ samples, data analysis, and interpretation. W. S participated in the reviewing of the manuscript. D. A. H and P. W. A. participated in the interpretation of data and reviewed and revised the manuscript. A. S. A participated in the study concept and design, interpretation of data, and reviewed and revised the manuscript. All authors read and approved the final manuscript.

## Funding

The work was supported by the Swedish National Centre for Sports Research (2024–0029), the Swedish Rheumatism Association (R‐1012837), and the Ulla Hamberg Angeby and Lennart Angeby Foundation (2023–02649, 2024–03910) to ASA. The funding agencies had no role or influence over or took part in the study design, data collection, analysis, interpretation of data, manuscript writing, or in the decision to submit the manuscript for publication.

## Consent

All the co‐authors give their consent for the findings related to this manuscript to be published.

## Conflicts of Interest

The authors declare no conflicts of interest.

## Supporting information


**Table S1:** Descriptive data for study subjects osteoarthritis (OA) patients included in study for Western blot analysis. Data presented as mean ± standard deviation. Related to Figures 1 and 2 (see also Material and Methods).

**Table S2:** Descriptive data for Osteoarthritis (OA) patients included in study for WB analysis. Related to Figures 3, and methods.

**Figure S1:** Western blot analysis of UBE2N, MGST1, PSMA and beta actin in lateral and medial menisci of OA seven Patients (P). L = lateral menisci, M = medial meniscus, LG = left leg, RT = right leg.

## Data Availability

The data used in this study are publicly accessible. The RNA‐seq dataset is available through the GEO database under accession number GSE185064, GSE263210, GSE19060, and GSE98918 (https://www.ncbi.nlm.nih.gov/geo/).

## References

[1] W. Jiang , H. Chen , Y. Lin , et al., “Mechanical Stress Abnormalities Promote Chondrocyte Senescence–The Pathogenesis of Knee Osteoarthritis,” Biomedicine & Pharmacotherapy 167 (2023): 115552. 37748410 10.1016/j.biopha.2023.115552

[2] K. Daszkiewicz and P. Luczkiewicz , “Biomechanics of the Medial Meniscus in the Osteoarthritic Knee Joint,” PeerJ 9 (2021): e12509. 34900428 10.7717/peerj.12509 PMC8627128

[3] C. Sheng , X. Zhou , J. Wang , et al., “Jiawei Yanghe Decoction Alleviates Osteoporotic Osteoarthritis by Promoting MSC Osteogenic Differentiation and Homing via ITGB6/TGF‐Beta/CXCR4 Pathway,” Phytomedicine 147 (2025): 157203. 40929885 10.1016/j.phymed.2025.157203

[4] X. Zhao , H. Xia , Y. Yang , et al., “Amygdalin and Magnesium Ions Exert Synergistic Effects on Cartilage Regeneration by Inhibiting Chondrocyte Ferroptosis via the IL‐17/GPX4 Axis,” J Orthop Translat 53 (2025): 246–259. 40678610 10.1016/j.jot.2025.05.006 PMC12269578

[5] P. F. Wong and T. Kamarul , “Targeting Ubiquitin‐Proteasome System (UPS) in Treating Osteoarthritis,” European Journal of Pharmacology 989 (2025): 177237. 39732357 10.1016/j.ejphar.2024.177237

[6] H. Y. Zhang , Z. Yao , L. Liu , et al., “Mechanical Overloading Promotes Chondrocyte Senescence and Osteoarthritis Development Through Downregulating FBXW7,” Annals of the Rheumatic Diseases 81, no. 5 (2022): 676–686. 35058228 10.1136/annrheumdis-2021-221513

[7] A. S. Ahmed , J. Li , H. Erlandsson‐Harris , A. Stark , G. Bakalkin , and M. Ahmed , “Suppression of Pain and Joint Destruction by Inhibition of the Proteasome System in Experimental Osteoarthritis,” Pain 153, no. 1 (2012): 18–26. 22018973 10.1016/j.pain.2011.08.001

[8] J. A. Bolduc , J. A. Collins , and R. F. Loeser , “Reactive Oxygen Species, Aging and Articular Cartilage Homeostasis,” Free Radical Biology and Medicine 132 (2019): 73–82. 30176344 10.1016/j.freeradbiomed.2018.08.038 PMC6342625

[9] M. L. Tiku , G. T. Allison , and R. Shah , “Chondrocyte Lipid Peroxidation (LP) Mediates Collagen Degradation: An In‐Vivo Role in Osteoarthritis?,” Arthritis and Rheumatism 43, no. 9 (2000): S351.

[10] Z. Jiang , D. Xue , X. Wen , et al., “Whole‐Transcriptome Sequence of Degenerative Meniscus Cells Unveiling Diagnostic Markers and Therapeutic Targets for Osteoarthritis,” Frontiers in Genetics 12 (2021): 754421. 34721542 10.3389/fgene.2021.754421 PMC8554121

[11] J. Zhang , J. Zhu , X. Zou , et al., “Identifying Autophagy‐Related mRNAs and Potential ceRNA Networks in Meniscus Degeneration Based on RNA Sequencing and Experimental Validation,” Heliyon 10, no. 12 (2024): e32782. 38975204 10.1016/j.heliyon.2024.e32782 PMC11226846

[12] J. T. Leek , W. E. Johnson , H. S. Parker , A. E. Jaffe , and J. D. Storey , “The Sva Package for Removing Batch Effects and Other Unwanted Variation in High‐Throughput Experiments,” Bioinformatics 28, no. 6 (2012): 882–883. 22257669 10.1093/bioinformatics/bts034 PMC3307112

[13] W. Tianzhi , H. Erqiang , X. Shuangbin , et al., “clusterProfiler 4.0: A Universal Enrichment Tool for Interpreting Omics Data,” Innovation 2, no. 3 (2021): 100141. 34557778 10.1016/j.xinn.2021.100141 PMC8454663

[14] P. Langfelder and S. Horvath , “WGCNA: An R Package for Weighted Correlation Network Analysis,” BMC Bioinformatics 9 (2008): 9. 19114008 10.1186/1471-2105-9-559 PMC2631488

[15] Y. Sun , D. R. Mauerhan , P. R. Honeycutt , et al., “Analysis of Meniscal Degeneration and Meniscal Gene Expression,” BMC Musculoskeletal Disorders 11 (2010): 19. 20109188 10.1186/1471-2474-11-19 PMC2828422

[16] R. H. Brophy , B. Zhang , L. Cai , R. W. Wright , L. J. Sandell , and M. F. Rai , “Transcriptome Comparison of Meniscus From Patients With and Without Osteoarthritis,” Osteoarthritis and Cartilage 26, no. 3 (2018): 422–432. 29258882 10.1016/j.joca.2017.12.004 PMC6007850

[17] S. L. Kolasinski , T. Neogi , M. C. Hochberg , et al., “2019 American College of Rheumatology/Arthritis Foundation Guideline for the Management of Osteoarthritis of the Hand, Hip, and Knee,” Arthritis Care & Research 72, no. 2 (2020): 149–162. 31908149 10.1002/acr.24131 PMC11488261

[18] E. Folkesson , B. Niederdorfer , V. T. Nakstad , et al., “High‐Throughput Screening Reveals Higher Synergistic Effect of MEK Inhibitor Combinations in Colon Cancer Spheroids,” Scientific Reports 10, no. 1 (2020): 11574. 32665693 10.1038/s41598-020-68441-0 PMC7360566

[19] J. Chen , J. Wang , W. Xinjie , et al., “eEF2 Improves Dense Connective Tissue Repair and Healing Outcome by Regulating Cellular Death, Autophagy, Apoptosis, Proliferation and Migration,” Cellular and Molecular Life Sciences 80, no. 5 (2023): 128. 37084140 10.1007/s00018-023-04776-x PMC10121543

[20] Q. He , Y. Lin , B. Chen , et al., “Vitamin K2 Ameliorates Osteoarthritis by Suppressing Ferroptosis and Extracellular Matrix Degradation Through Activation GPX4's Dual Functions,” Biomedicine & Pharmacotherapy 175 (2024): 116697. 38759289 10.1016/j.biopha.2024.116697

[21] N. Jiang , B. Xing , R. Peng , et al., “Inhibition of Cpt1a Alleviates Oxidative Stress‐Induced Chondrocyte Senescence via Regulating Mitochondrial Dysfunction and Activating Mitophagy,” Mechanisms of Ageing and Development 205 (2022): 111688. 35728631 10.1016/j.mad.2022.111688

[22] F. Kuang , J. Liu , Y. Xie , et al., “MGST1 Is a Redox‐Sensitive Repressor of Ferroptosis in Pancreatic Cancer Cells,” Cell Chemical Biology 28, no. 6 (2021): 765–775.e5. 33539732 10.1016/j.chembiol.2021.01.006

[23] D. Kang , J. Lee , J. Jung , et al., “Selenophosphate Synthetase 1 Deficiency Exacerbates Osteoarthritis by Dysregulating Redox Homeostasis,” Nature Communications 13, no. 1 (2022): 779. 10.1038/s41467-022-28385-7 PMC8828855 35140209

[24] R. Morgenstern , J. Zhang , and K. Johansson , “Microsomal Glutathione Transferase 1: Mechanism and Functional Roles,” Drug Metabolism Reviews 43, no. 2 (2011): 300–306. 21495795 10.3109/03602532.2011.558511

[25] M. Sobczak , T. Boczek , A. Kowalski , M. Wiktorska , J. Niewiarowska , and L. Zylinska , “Downregulation of Microsomal Glutathione‐S‐Transferase 1 Modulates Protective Mechanisms in Differentiated PC12 Cells,” Journal of Physiology and Biochemistry 70, no. 2 (2014): 375–383. 24419913 10.1007/s13105-014-0312-9

[26] J. J. Lenoir , J. P. Parisien , and C. M. Horvath , “Immune Regulator LGP2 Targets Ubc13/UBE2N to Mediate Widespread Interference With K63 Polyubiquitination and NF‐KB Activation,” Cell Reports 37, no. 13 (2021): 110175. 34965427 10.1016/j.celrep.2021.110175 PMC8787715

[27] F. Liu , H. Yang , T. Yang , et al., “Dysregulated Proteasome Activity and Steroid Hormone Biosynthesis Are Associated With Mortality Among Patients With Acute COVID‐19,” Journal of Translational Medicine 22, no. 1 (2024): 626. 38965561 10.1186/s12967-024-05342-0 PMC11229496

[28] K. S. Emanuel , L. Huang , M. J. J. Haartmans , et al., “Patient‐Responsive Protein Biomarkers for Cartilage Degeneration and Repair Identified in the Infrapatellar Fat Pad,” Expert Review of Proteomics 21, no. 12 (2024): 563–573. 10.1080/14789450.2024.2438774 39635821

[29] S. Liao , Q. Zheng , H. Shen , et al., “HECTD1‐Mediated Ubiquitination and Degradation of Rubicon Regulates Autophagy and Osteoarthritis Pathogenesis,” Arthritis and Rheumatology 75, no. 3 (2023): 387–400. 36121967 10.1002/art.42369

[30] G. Wang , S. Chen , Z. Xie , et al., “TGFβ Attenuates Cartilage Extracellular Matrix Degradation via Enhancing FBXO6‐Mediated MMP14 Ubiquitination,” Annals of the Rheumatic Diseases 79, no. 8 (2020): 1111–1120. 32409323 10.1136/annrheumdis-2019-216911 PMC7392491

[31] Y. A. Li , S. J. Li , and H. J. Wu , “Ubiquitination‐Proteasome System (UPS) and Autophagy Two Main Protein Degradation Machineries in Response to Cell Stress,” Cells 11, no. 5 (2022): 851. 35269473 10.3390/cells11050851 PMC8909305

