# Supplementary material for: Network Analysis Identifies Microsomal Glutathione S‐Transferase as a Potential Regulator of Oxidative Stress and Proteasome Dysfunction in Human Osteoarthritic Menisci
Source: FASEB Bioadv. 2026 Apr 27;8(5):e70101. doi: 10.1096/fba.2025-00302 (PMC13111912; doi:10.1096/fba.2025-00302)
Supplement: Supplementary file 1 — Table S1: Descriptive data for study subjects osteoarthritis (OA) patients included in study for Western blot analysis. Data presented as mean ± standard deviation. Related to Figures 1 and 2 (see also Material and Methods). [file FBA2-8-e70101-s003.docx]

**Supplementary Table 1.** Descriptive data for study subjects osteoarthritis (OA) patients included in study for Western blot analysis. Data presented as mean ± standard deviation. Related to Figure 1 and 2 (see also Material and Methods).

|  | **GSE185064** | | **GSE263210** | | **GSE19060** | | **GSE98918** | |
| --- | --- | --- | --- | --- | --- | --- | --- | --- |
|  | **OA** | **Control** | **OA** | **Control** | **OA** | **Control** | **OA** | **Control** |
| **Study subjects (N)** | 5 | 4 | 5 | 4 | 5 | 3 | 12 | 12 |
| **Age, median (range)** | 67.26  (50-80) | 38.75  (18-50) | 76  (69-82) | 25  (20-36) | 56  (50-65) | 39  (12-43) | 53  (31-65) | 64  (53-80) |
| **Sex male/female (N)** | 1/4 | 3/1 | 2/3 | 1/3 | 1/4 | 2/1 | 3/9 | 6/6 |
