# Supplementary material for: Network Analysis Identifies Microsomal Glutathione S‐Transferase as a Potential Regulator of Oxidative Stress and Proteasome Dysfunction in Human Osteoarthritic Menisci
Source: FASEB Bioadv. 2026 Apr 27;8(5):e70101. doi: 10.1096/fba.2025-00302 (PMC13111912; doi:10.1096/fba.2025-00302)
Supplement: Supplementary file 2 — Table S2: Descriptive data for Osteoarthritis (OA) patients included in study for WB analysis. Related to Figures 3, and methods. [file FBA2-8-e70101-s001.docx]

**Supplementary Table 2.** Descriptive data for Osteoarthritis (OA) patients included in study for WB analysis. Related to Figures 3, and methods.

| **Samples** | **Age** | **Sex** | **Tissues (Menisci)** | |
| --- | --- | --- | --- | --- |
| Patient 1 OA | 56 | M | Lateral |  |
| Patient 2 OA | 69 | F | Lateral | Medial |
| Patient 3 OA | 79 | F | Lateral | Medial |
| Patient 4 OA | 72 | M | Lateral | Medial |
| Patient 5 OA | 64 | M | Lateral | Medial |
| Patient 6 OA | 63 | M | Lateral | Medial |
| Patient 7 OA (Left Leg) | 66 | M | Lateral | Medial |
| Patient 7 OA (Right leg) | 66 | M | Lateral | Medial |
