# Supplementary material for: Network Analysis Identifies Microsomal Glutathione S‐Transferase as a Potential Regulator of Oxidative Stress and Proteasome Dysfunction in Human Osteoarthritic Menisci
Source: FASEB Bioadv. 2026 Apr 27;8(5):e70101. doi: 10.1096/fba.2025-00302 (PMC13111912; doi:10.1096/fba.2025-00302)

**Supplementary Figure 1.** Western blot analysis of UBE2N, MGST1, PSMA and beta actin in lateral and medial menisci of OA seven Patients (P). L = lateral menisci, M = medial meniscus, LG = left leg, RT = right leg.


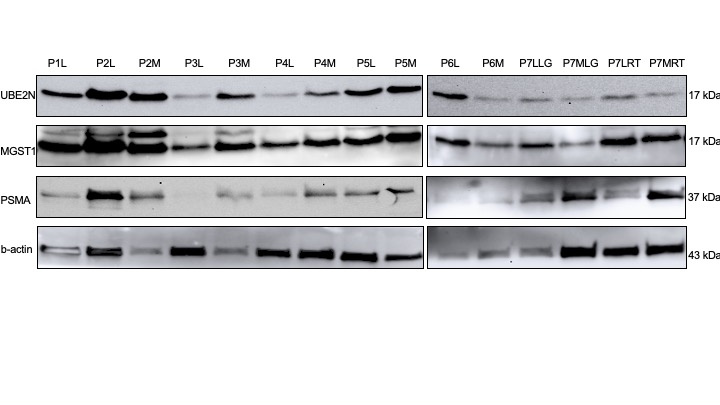

Supplement: Supplementary file 3 — Figure S1: Western blot analysis of UBE2N, MGST1, PSMA and beta actin in lateral and medial menisci of OA seven Patients (P). L = lateral menisci, M = medial meniscus, LG = left leg, RT = right leg. [file FBA2-8-e70101-s002.docx]
